# Conus medullaris syndrome due to an intradural disc herniation: A case report

**DOI:** 10.4103/0019-5413.38590

**Published:** 2008

**Authors:** Kshitij S Chaudhary, Mihir R Bapat

**Affiliations:** Department of Orthopaedics, KEM Hospital, Mumbai, India

**Keywords:** Conus medullaris syndrome, intradural disc herniation

## Abstract

A 70-year-old male patient developed acute paraplegia due to conus medullaris compression secondary to extrusion of D12-L1 disc. After negative epidural examination intraoperatively, a durotomy was performed and an intradural disc fragment was excised. Patient did not regain ambulatory status at two-year follow-up. Intraoperative finding of negative extradural compression, tense swollen dura and CSF leak from ventral dura should alert the surgeon for the possibility of intradural disc herniation. A routine preoperative MRI is misleading and a high index of suspicion helps to avoid a missed diagnosis.

## INTRODUCTION

Intradural migration of a herniated intervertebral disc is an extremely rare and a potentially catastrophic complication. The incidence reported varies between 0.04-0.33%.^1^ We hereby present one such case.

A 70-year-old patient presented with sudden onset acute flaccid paraplegia of six hours duration. He had experienced a sharp stabbing pain in the mid-dorsal spine that radiated along the lower ribs, while attempting to pick up an object form the ground. The intensity of pain had spontaneously and significantly reduced over six hours. The patient had been a manual laborer lifting heavy weights for 30 years but had led a retired sedentary lifestyle for the last two years. He had never received treatment for significant back pain. Palpation of the spine revealed no deformity, tenderness or muscle spasm. The patient was unable to move both lower limbs voluntarily. There was significant hypoesthesia below the groin crease. The deep tendon reflexes were absent in both lower extremities. He was catheterized on admission to decompress an insensate and distended bladder.

**Figure 1:**
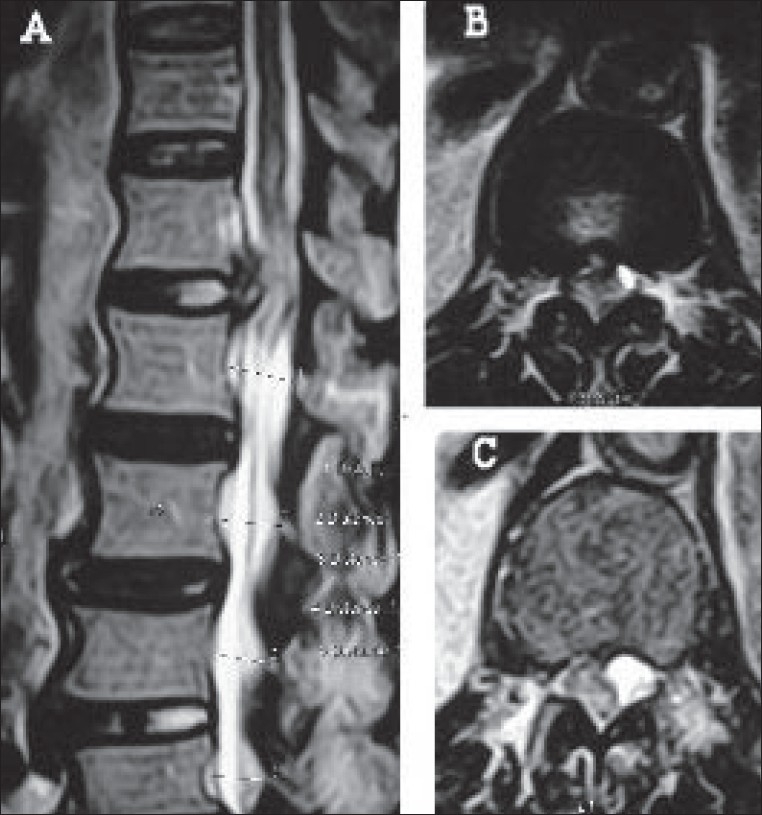
T2W MRI sagittal (A) and axial images (B and C) showing disc herniation at D12-L1. Disc fragment lying beneath the D12 lamina displacing conus antero-laterally and cord edema (C)

Magnetic resonance imaging was performed 10 hrs after the episode. A right paracentral extrusion of the D12-L1 disc was noticed with compression of the conus medullaris [Figure 1]. A sequestrated fragment had displaced the conus to the left and appeared to lie beneath the lamina of the D12 vertebra. Cord edema was noticed as a hyperintense signal within the cord substance on the T2 weighted MRI image [Figure 1]. A concomitant stenosis was present at the L4-L5 level. A conus medullaris syndrome due to extradural compression from a herniated D12-L1 disc was diagnosed and an urgent decompression was done at 16 hrs after presentation. No steroids were administered prior to surgery.

Considering the location of the sequestrated disc fragment a posterior approach was preferred. A D12 laminectomy was performed. No extradural disc fragment was visualized. The dural sac was not displaced, but appeared swollen. On gently retracting the dura towards the left, cerebrospinal fluid leaked from the anterior unseen portion of the dura. At this juncture an intradural herniation was first suspected and durotomy performed. A solitary large desiccated fragment was visualized. The ventral dural rent had ragged edges and was adherent to the posterior annulus and the posterior longitudinal ligament. The disc fragment was teased off the cord substance that was displaced antero-laterally. A small piece of gel foam was placed over the anterior dural defect and the posterior durotomy repaired. Decompression of the coexistent L4-L5 stenosis was carried out because patient had given a history of claudicating leg pains prior to this episode, for a duration of two years, though these symptoms were mild to moderate.

The patient recovered normal sensations by the third postoperative day. Bladder control was achieved on the fifth day after surgery. At two years follow-up the patient had normal sensations and voided normally but complained of urge incontinence. Motor recovery started in the first postoperative week in the right knee extensor (Grade 2 MRC scale) and then exhibited a long plateau phase till the fifth postoperative week. The recovery then progressed till six months in both lower extremities but did not reach an ambulatory status (Grade 2 MRC scale) at the final two-year follow-up. The anterior dural rent healed uneventfully.

**Figure 2:**
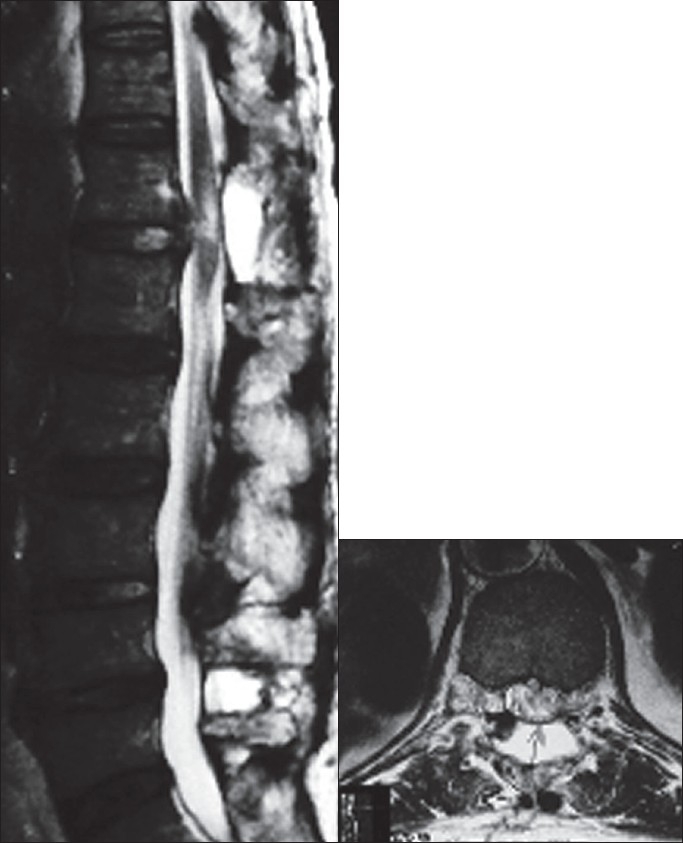
T2W MRI sagittal and axial images at 1 year followup showing expansion of cord and myelomalacia

Histopathology revealed a degenerated disc fragment with no evidence of intra-substance calcification. An MRI performed at one year showed expansion of the cord with myelomalacia evident on T2 weighted images [Figure 2].

## DISCUSSION

We hereby present one such case of the 278 degenerative lumbar procedures performed between 2001 and 2005 by the author; this is the first case of an intradural disc herniation. Average age of presentation is higher than the extradural disc ruptures, presenting usually in the fifth and the sixth decade. Males represent 76% of the reported cases.^1^

In 95%, the lumbar spine is affected and the commonest levels are L3-4 and L4-5. The incidence of intradural herniation decreases as one proceeds towards the lumbosacral junction. It is suggested that, narrower lumbar canal at higher levels may predispose for a higher lumbar localization of intradural herniations.^1^ Rarely, intradural migration in the cervical and the dorsal spine has been reported to produce compressive myelopathy. To our knowledge, D12-L1 intradural disc herniation causing conus medullaris syndrome has not been reported in the literature.

The pathogenesis of intradural herniation is not known with certainty. It is unclear whether an acute disc herniation can rupture the dura. In most instances the erosion may be precipitated by, 1. intradiscal calcification, 2. attrition of the ventral dura due to previous herniation, 3. adhesions between the dura and the posterior longitudinal ligament that may be congenital or acquired during repeated herniation or surgery^2^,^3^ and 4. spinal canal stenosis.^4^ Adhesions and spinal canal stenosis with superimposed acute disc herniations was probably responsible for the occurrence of intradural migration in this case.

There are no differentiating clinical signs between an extradural and intradural disc herniation, though the severity of neurological deficit is higher in the later group.^4^,^5^ A polyradiculopathy with affection of the traversing as well as the exiting nerve roots should alert the surgeon towards this rare event, especially in disc herniation at higher levels in the lumbar spine. The incidence of cauda equina syndrome is significantly higher (33%) with intradural disc herniation as compared to extradural herniation (less than one per cent) that occurs at higher levels in the lumbar spine.^5^

The urge to perform emergency decompression in the face of severe neurological deficit and absence of pathognomonic signs on the preoperative plain magnetic resonance imaging, is responsible for a missed diagnosis in almost all cases. As most authors emphasize, the intradural migration becomes apparent during surgery when extradural compression is conspicuously absent. A tense swollen dura is the only tell-tale sign. Cerebrospinal fluid (CSF) leak from the anterior aspect of the dura is sealed due to adhesions between the dura and the posterior longitudinal ligament and becomes apparent only on probing.^6^ Intraoperative myelogram and ultrasonography act as adjuncts to a conclusive negative intraoperative examination.^7^ Before durotomy it is always advisable to confirm the level by radiographs to avoid a wrong level exploration

In suspicious cases, a gadolinium contrast enhanced MRI helps to diagnose and differentiate an intradural disc fragment from other mass lesions.^8^ An intradural fragment at the level of the disc space with enhancement extending from the disc space to the fragment suggests the diagnosis.^8^ While a contrast MRI may be warranted in chronic intradural mass lesions or missed lesions that require revision surgery, it is debatable whether all patients of acute disc herniation should be subjected to the contrast MRI in view of rarity of presentation. Acute intradural ruptures may not enhance with gadolinium due to avascularity of the sequestrated fragment.^9^ Delayed revascularization by epidural vessels allows enhancement in chronic lesions.^8^

In acute lesions, neurological recovery is satisfactory after excision of the fragment. The factors that adversely affect the prognosis are the chronicity and severity of preoperative neurological deficit.^3^,^10^ Delay in surgical decompression and presence of cauda equina syndrome seems to have a negative impact on surgical outcome.^1^,^10^

A diligent search based on high index of suspicion for retained intradural fragment helps to avoid revision surgery for missed diagnosis.
